# Clinical Outcome of Intraoperative Recurrent Laryngeal Nerve Monitoring during Thoracoscopic Esophagectomy and Mediastinal Lymph Node Dissection for Esophageal Cancer

**DOI:** 10.3390/jcm11174949

**Published:** 2022-08-23

**Authors:** Chang-Lun Huang, Chun-Min Chen, Wei-Heng Hung, Ya-Fu Cheng, Ruei-Ping Hong, Bing-Yen Wang, Ching-Yuan Cheng

**Affiliations:** 1 Division of Thoracic Surgery, Department of Surgery, Changhua Christian Hospital, Changhua 500, Taiwan; 2 Graduate Institute of Biomedical Science, China Medical University, Taichung 404, Taiwan; 3 Big Data Center, Changhua Christian Hospital, Changhua 500, Taiwan; 4 Department of Post-Baccalaureate Medicine, College of Medicine, National Chung Hsing University, Taichung 407, Taiwan; 5 School of Medicine, Chung Shan Medical University, Taichung 402, Taiwan; 6 School of Medicine, College of Medicine, Kaohsiung Medical University, Kaohsiung 807, Taiwan; 7 Institute of Genomics and Bioinformatics, National Chung Hsing University, Taichung 402, Taiwan; 8 Center for General Education, Ming Dao University, Changhua 523, Taiwan

**Keywords:** esophageal cancer, esophagectomy, intraoperative nerve monitoring, recurrent laryngeal nerve, vocal cord palsy

## Abstract

Mediastinal lymph dissection in esophagectomy for patients with esophageal cancer is important. The dissection of recurrent laryngeal nerve (RLN) lymph nodes could cause RLN injury, vocal cord palsy, pneumonia, and respiratory failure. This retrospective study aimed to evaluate the effects of intraoperative RLN monitoring in esophagectomy and mediastinal lymph node dissection in preventing RLN injury and vocal cord palsy. This study included 75 patients who underwent minimally invasive esophagectomy and mediastinal lymph node dissection for esophageal cancer with (38 patients) and without (37 patients) IONM at Changhua Christian Hospital from 2015 to 2020. The surgical and clinical outcomes were reviewed. Patients in the IONM group had more advanced clinical T status, shorter operation time (570 vs. 633 min, *p* = 0.007), and less blood loss (100 mL vs. 150 mL, *p* = 0.019). The IONM group had significantly less postoperative vocal palsy (10.5% vs. 37.8%, *p* = 0.006) and pneumonia (13.2% vs. 37.8%, *p* = 0.014) than that in the non-IONM group. IONM was an independent factor for less postoperative vocal cord palsy that was related to postoperative 2-year survival. This study demonstrated that IONM could reduce the incidence of postoperative vocal cord palsy and pneumonia.

## 1. Introduction

Esophageal cancer is the sixth most reported lethal malignancy and accounts for approximately 5.5% of all cancer deaths worldwide [1]. Esophagectomy remains the main curative treatment for resectable esophageal cancer in the multi-modality treatment era [2]. In addition, mediastinal lymph node dissection is an important procedure during esophagectomy, which may offer precise staging and survival benefits [3,4,5]. Squamous cell carcinoma (SCC) and adenocarcinoma (AC) of the esophagus demonstrate different behaviors, risk factors, tumor locations, prognosis, and so on. However, the lymph node metastasis pattern over the mediastinum of AC is similar and even more aggressive than SCC [6,7,8]. Adequate lymph node dissection is important in both SCC and AC. Recurrent laryngeal nerve (RLN) lymph node is one of the important upper mediastinal lymph node groups during radical lymph node dissection. The metastasis rate of RLN lymph nodes was reported to be between 27% and 43.3% [9,10,11], which is higher in upper thoracic esophageal squamous cell carcinoma. Metastatic RLN lymph nodes indicate poor prognosis and could be a predictor for cervical lymph node involvement. Vocal cord paresis or paralysis caused by RLN injury is one of the critical complications during radical lymph node dissection. Clinical manifestations of RLN injury include hoarseness and subjective choking, which may result in pneumonia, respiratory failure, and the need for tracheostomy [12,13,14,15]. Intraoperative RLN monitoring has been proven to reduce the incidence of RLN paralysis during thyroid surgery [16], but the result in mediastinal lymph node dissection during esophagectomy is equivocal. Thus, this study aimed to evaluate the efficacy of intraoperative RLN monitoring to reduce nerve injury and its complications in patients undergoing esophagectomy and mediastinal lymph node dissection for resectable thoracic esophageal cancer.

## 2. Patients and Methods

### 2.1. Patients

This study included 75 patients who underwent minimally invasive esophagectomy and gastric tube reconstruction for thoracic esophageal cancer from 2015 to 2020. All operations were performed by the same surgical team in our institution led by surgeon C.Y.C. All patients were diagnosed with thoracic esophageal cancer by endoscopic biopsy. Staging workup included enhanced chest computed tomography, positron emission tomography/computed tomography scan, whole-body bone scan, brain magnetic resonance imaging, liver sonography, and bronchoscopy. Patients underwent upfront surgery or neoadjuvant concurrent chemoradiation therapy (CCRT) followed by surgery according to clinical T and N status after our multidisciplinary team discussion. Patients with preoperative hoarseness or vocal cord paresis proven by bronchoscopy or those who underwent concurrent laryngectomy for head and neck cancer were excluded. Postoperative hoarseness and vocal cord palsy were detected according to clinical symptoms and signs and confirmed with bronchoscopy. Prolonged air leakage was defined as persistent air leakage from the chest drain for >5 days. Anastomotic leakage was detected with foamy discharge from the cervical drain at the esophagogastrostomy site and confirmed with esophagoscopy or esophagography. Patients with body temperature >38.3 °C, leukocytosis, infiltration on chest X-ray, and positive sputum culture were defined as pneumonia, and appropriate antibiotics treatment was initiated after detection. In 2014, intraoperative neuromonitoring (IONM) for RLN during esophagectomy was introduced in our hospital but was not applied to every patient due to financial factors.

### 2.2. Intubation and Anesthesia

General anesthesia with one-lung ventilation was administered to all patients. Patients with IONM were intubated with an 8.0 mm Medtronic NIM TriVantage™ EMG endotracheal tube. The tube had two exploratory electrodes above the cuff, and its position was confirmed by anesthetists with a laryngoscope during intubation. An endobronchial blocker was then placed to achieve lung isolation during the operation. Patients without IONM were intubated with a double-lumen endotracheal tube or an 8.0 mm single-lumen endotracheal tube with an endobronchial blocker. A short-acting muscle relaxant was used to induce and maintain anesthesia. The muscle relaxant was ceased at least 30 min before performing the mediastinal lymph node dissection to restore muscle activity and minimize its signal detection effect.

### 2.3. Surgical Procedure

We performed modified McKeown minimally invasive esophagectomy (MIE) with two-field lymph node dissection and left cervical lymph node sampling in all patients. Patients were placed in a semi-prone position after general anesthesia and intubation. Thoracoscopic esophagectomy and mediastinal lymph node dissection were performed with four trocars and carbon dioxide insufflation at 6 mmHg. Right and left RLN lymph node groups were identified and harvested with or without IONM. Endoscopic scissors and energy devices, such as harmonic scalpel, were used during lymph node dissection but were kept away from the expected RLN location, which was indicated by the nerve monitor. Patients were re-positioned to a supine position, a laparoscopic gastric conduit was created, and upper abdominal lymphadenectomy was performed. A left neck incision was made, and the esophagus and gastric conduit were retrieved upward. Esophagogastrostomy was performed with a hand-sewn or circular stapler method, depending on the surgeon’s preference.

### 2.4. Intraoperative Monitoring for RLN

The two electrodes in the EMG tube were connected to the NIM nerve monitoring system, which continuously monitors EMG activity from the vocal cords innervated by the RLN. We used a commercial hand-held nerve stimulator with initial electric currents of 1.0 mA as the starting point, and the event threshold was set at 100 μV. The vagus nerve was identified and appropriately skeletonized after opening the upper mediastinal pleura. A nerve stimulator was used at the upper third vagus nerve and moved upward to the subclavian artery before harvesting the lymph node. The current was elevated to 2.0 mA and up to 3.0 mA if a suboptimal response was noted. Once the right RLN was identified, careful dissection was performed, and the heat effect of the energy device was avoided to the nerve. The nerve stimulator was used to identify left RLN before harvesting the subaortic lymph node group. Lymph nodes along the left paratracheal groove were also examined with nerve monitoring guidance. Both nerve activities were reconfirmed after completing the lymph node dissection. During the neck procedure, nerve stimulator and response monitoring were used before and after lymph node sampling.

### 2.5. Follow-up and Data Collection

A retrospective chart review was conducted using a standardized outcome protocol. Data were collected regarding patient demographics, surgical procedures and outcomes, and the use of IONM. The primary outcome includes postoperative vocal cord palsy and pneumonia. This study considered all causes of mortality. Failure to recover the vocal cord function 6 months postoperatively was defined as permanent laryngeal nerve palsy. All patients were regularly evaluated at the outpatient department after discharge. Follow-up data were collected by chart review, and evaluations were completed every 3 months in the first and second years postoperatively. The study was conducted in accordance with the Declaration of Helsinki and was approved by the Ethics Committee of Changhua Christian Hospital, Taiwan (IRB-210316).

### 2.6. Statistical Analysis

All analyses were conducted in Statistical Package for the Social Sciences version 22.0 (IBM Corporation, Armonk, NY, USA). Comparisons of demographic and clinical characteristics were performed with the Mann–Whitney U test for continuous variables and chi-square tests or Fisher’s exact test for categorical variables. The 2-year survival rate was estimated with a Kaplan–Meier survival analysis. A Cox proportional hazards model was used to evaluate the effects of potential predictors on outcome events (death). Predictor variables were generated by the surgical outcomes, e.g., vocal cord palsy, operation time, blood loss, complications, pT stage, pN stage, etc., and clinical characteristics included tumor location, cell type, neoadjuvant CCRT, history of head and neck cancer, intensive care unit (ICU) duration, and hospital stay. Demographic variables were also included (age and gender). Backward elimination was adopted for variable selection methods. The method begins with a full model, including all the possible explanatory variables, and removes insignificant variables from the model [17]. Statistical significance was defined as a two-sided *p*-value of <0.05.

## 3. Results

### 3.1. Patient Clinical Data

This study included 75 patients (68 males and 7 females, aged between 33 and 81 years, median age: 59 years). Of the patients, 38 underwent MIE with IONM during mediastinal lymph node dissection (IONM group) and 37 underwent MIE without IONM (non-IONM group). Table 1 demonstrates patient demographics. More advanced clinical T stage was observed in the IONM group (patients with clinical stages 3 and 4 were 60% and 38% in the IONM and non-IONM groups, respectively). There were no significant differences in age, gender, history of head and neck cancer, neoadjuvant chemoradiation, clinical N stage, tumor location, and cell type.

### 3.2. Surgical Outcomes

Table 2 details the surgical outcomes between the IONM and non-IONM groups. The IONM group had a shorter operation time (570 vs. 633 min, *p* = 0.007) and less blood loss (100 mL vs. 150 mL, *p* = 0.019) than the non-IONM group. The differences in ICU and total length of hospital stay were not statistically significant. The pathological T and N stages were also similar in both groups. The harvested lymph nodes revealed no significant difference in terms of harvested mediastinal or total lymph nodes. Harvested right RLN lymph nodes were similar in both groups, but more left RLN lymph nodes were dissected in the IONM group (3.1 ± 3.2 vs. 1.8 ± 2.0, *p* = 0.043).

### 3.3. Postoperative Morbidity and Survival Outcome

No surgical mortality (death within 30 days postoperatively) occurred in either group. The postoperative morbidity between groups is summarized in Table 3. Postoperative vocal cord palsy was more frequently observed in the non-IONM group than in the IONM group (37.8% vs. 10.5%, *p* = 0.006), and left side vocal cord palsy was much more observed in the non-IONM group (non-IONM group: 10/14, 71.4% and IONM group: 2/4, 50%). There was no difference between the two groups in terms of anastomotic leakage and other morbidities, except pneumonia, which was much higher in the non-IONM group (37.8% vs. 13.2%, *p* = 0.014).

**Figure 1:**
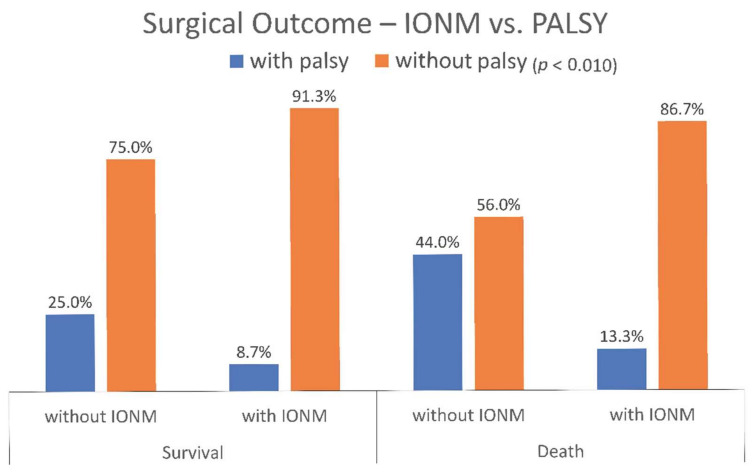
Percentage of IONM use and palsy observed by survival status.

Figure 1 further presents the relationship between IONM use and postoperative vocal cord palsy according to survival status. In the survival group, 91% of patients in the IONM group had no postoperative vocal cord palsy, whereas 25% of those without IONM had. In the death group, 87% of patients in the IONM group did not develop postoperative vocal cord palsy, while nearly 44% of non-IONM recipients did. A lower incidence of postoperative vocal cord palsy was found in patients treated with IONM, suggesting IONM therapy could benefit postoperative vocal cord palsy patients.

#### Survival Outcome

**Figure 2:**
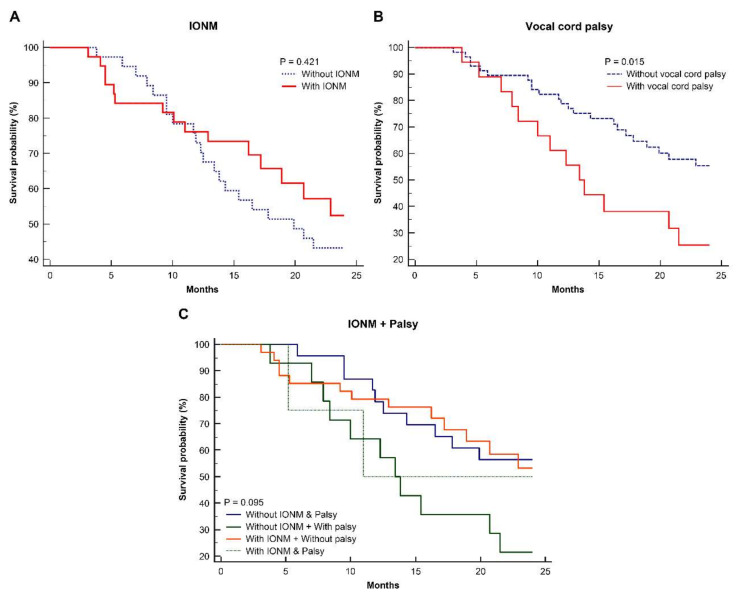
Two-year survival in patients with thoracic esophageal cancer between those (**A**) with and without IONM, (**B**) with and without vocal cord palsy, and (**C**) with or without IONM + palsy.

This study revealed a median follow-up period of 17.2 months. The Kaplan–Meier curves showed that IONM did not significantly affect the 2-year overall survival (OS) (Figure 2A). In contrast, patients without palsy had better 2-year OS than those with palsy (Figure 2B). A difference was found between the palsy groups; thus, subgroup analysis was performed according to IONM-palsy, as shown in Figure 2C. Results showed a higher survival rate for patients without vocal cord palsy, while those without IONM and with palsy had the worst survival rate, which approached marginal significance (*p =* 0.05–0.1).

### 3.4. Postoperative Vocal Cord Palsy Predictors and Influence of OS

IONM was the only factor that was left and significantly predicted palsy in the final backward elimination multivariable regression model adjusted for all possible variables (odds ratio [OR] = 0.193, 95% confidence interval [CI] = 0.06–0.66, *p* = 0.009) (data not shown).

The prognostic analysis of all clinical variables was conducted using Cox proportional hazards model. Table 4 lists the estimates from the final Cox regression model. Vocal cord palsy (hazard ratio [HR] = 2.98, 95% CI: 1.32–6.71; *p* = 0.008), age (HR = 1.05, 95% CI: 1.01–1.08; *p* = 0.016), pT (Tis + T1a/b + T2) (HR = 7.25, 95% CI: 2.09–25.17; *p* = 0.002), pT (T3 + T4) (HR = 12.53, 95% CI: 3.62–43.35; *p* < 0.000), neoadjuvant CCRT (HR = 8.37, 95% CI: 3.59–19.52; *p* < 0.000), and ICU duration (HR = 1.11, 95% CI: 1.00–1.2; *p* = 0.040) were prognostic predictors of OS after multivariate analysis.

## 4. Discussion

The protective effects of IONM during esophagectomy were diversely reported in previous studies [18,19,20]. Our study revealed a significant benefit of IONM on postoperative vocal cord palsy and pneumonia, as well as the relationship between IONM, vocal palsy, and mortality, wherein IONM may relate to prolonged 2-year survival after thoracoscopic esophagectomy for esophageal cancer. Vocal cord palsy after surgery, operation time, blood loss, and pneumonia were all significantly less in the IONM group than in the non-IONM group. More advanced clinical T stage and more harvested left RLN lymph nodes were observed in the IONM group. This study demonstrates that IONM can provide significant clinical benefit to patients with cancer undergoing thoracoscopic esophagectomy for esophageal cancer.

Esophagectomy with lymph node dissection is the mainstream curative treatment in patients with resectable thoracic esophageal cancer. Several studies disclosed that the extent of lymph node dissection was related to prognosis [3,4,5]. However, the increased extent of lymphadenectomy may be related to more postoperative morbidity [21]. In our study, two-field lymph node dissection with left cervical lymph node sampling was applied to all the patients. Total harvested lymph nodes were also similar in both groups (41.9 ± 13.9 in the IONM group and 41.5 ± 11.7 in the non-IONM group, *p* = 0.898). The importance of RLN lymph node during upper mediastinal lymph node dissection was strongly suggested as its high prevalence, being an indicator of cervical lymph node metastasis and prognostic factor of OS [10]. The metastasis rate of RLN lymph nodes was reported to be between 27% and 43.3% [9,10,11]. Our study revealed 19 patients (25.3%) with RLN lymph node metastasis and 2 with both right and left RLN lymph node metastasis. The nerve was susceptible to injury with mechanical stretch or thermal effect by electrocautery or heat produced by energy devices during the harvesting of RLN lymph nodes. RLN injuries may cause temporary or long-term vocal cord dysfunction. Vocal cord palsy may result in hoarseness, choking when swallowing, impaired secretion clearance ability, and aspiration pneumonia. Shimizu et al. performed subtotal esophagectomy in 125 patients and revealed 26 patients (20.8%) with grade II or more postoperative RLN paralysis [13]. Oshikiri et al. reported a 43% RLN injury in 209 patients undergoing MIE with 21% pneumonia [14]. Our study revealed 18 patients (24%) with postoperative RLN palsy and 19 (25.3%) with pneumonia. Adaption of appropriate techniques and delicate surgical maneuvers were necessary to prevent RLN injury during lymph node dissection.

IONM in thyroid surgery has been developed for decades and could decrease the incidence of RLN paralysis [16]. In 2001, Hemmerling et al. were the first to apply IONM for RLN in esophagectomy by attaching two electrodes around a double-lumen endotracheal tube [22]. Schmidt et al. then suggested a novel approach to use the commercial EMG endotracheal tube system, which was commonly used in thyroid surgery combined with an endobronchial blocker, to achieve single lung ventilation during esophageal surgery [23]. These became the standard in the following practice of IONM during esophagectomy. Several studies have demonstrated their experience with IONM in esophagectomy. Gelpke et al. shared their experience with 12 patients undergoing thoracic procedures and revealed the feasibility of identifying RLN during operation [24]. Zhong et al. summarized the results of IONM in 54 of 115 patients and revealed more vocal cord palsy and pneumonia in patients without IONM, as well as less positive and harvested mediastinal and total lymph nodes [18]. Hikage et al. applied IONM in patients undergoing prone esophagectomy and revealed 92.7% and 88% sensitivity for detecting the right and left RLN, respectively [19]. Three of their patients in the IONM group could not be successfully monitored for nerve activities due to muscle relaxant usage. Postoperative RLN palsy, aspiration, and pneumonia were similar in both groups. Takeda et al. revealed that IONM could decrease grade II or more RLN palsy but without difference in terms of pneumonia, anastomotic leakage, and other complications [20]. Our study revealed that all patients in the IONM group could complete the IONM with a positive signal before and after dissection. In addition, the voltage of nerve stimuli was necessary to be larger in patients with abundant fat pad and those with neoadjuvant chemoradiation. We minimized the usage of muscle relaxants and performed RLN lymph node dissection as the first step. Muscle relaxants could be administered after completing the RLN lymph node dissection and reconfirmation of nerve activity. Patients in our IONM group seemed to have more advanced clinical T status, probably from coincidence or selection bias by the surgeon and patient’s decision after shared decision-making. The operation time and blood loss were significantly less in the IONM group. This is thought to be caused by the identification of bilateral RLN during lymph node dissection. We still performed RLN lymph node dissection without IONM but took more time to visually confirm its possible location and avoid the use of the electrocautery of energy device for hemostasis.

Zhong et al. showed better lymph node dissection quality in patients with IONM [18], but the harvested right RLN, mediastinal, and total lymph nodes in our two groups were similar. This represented our equal effort for lymph node dissection over the RLN region and may result in longer operation time and more RLN injuries. However, left RLN lymph nodes were less harvested in the non-IONM group than in the IONM group (1.8 ± 2.0 vs. 3.1 ± 3.2, *p* = 0.043). These may be explained by the difficulty of lymph node dissection and nerve identification around the subaortic region and left tracheoesophageal groove without the IONM.

Postoperative vocal cord palsy developed in 4 patients (10.5%) in the IONM group and 14 (37.8%) in the non-IONM group in our study. The incidence of vocal cord palsy in our non-IONM group (37.8%) was similar to the results published by Hikage et al. (42.5%) [19], but patients in the IONM group had significantly less vocal cord palsy and pneumonia rate. Our study revealed more left-sided vocal cord palsy than right-sided in the non-IONM (10/14, 71.4%) compared to the IONM group (2/4, 50%), probably because the left RLN is much longer and is located lower and across the subaortic, left tracheobronchial, and left paratracheal lymph node territory. All these characteristics made the left RLN more susceptible to injury during lymph node dissection. The postoperative complications between the 2 groups showed no significant difference, except pneumonia, which developed in 5 patients in the IONM group and 14 in the non-IONM group (13.2% vs. 37.8%, *p* = 0.014), and these may correlate to vocal palsy. All patients, except one (in the non-IONM group), clinically improved after antibiotics use, with enhanced airway hygiene and pulmonary rehabilitation.

Zhong et al. reported that patients with IONM had a better 2-year survival rate [18]. Our study revealed that most patients in the survival group that underwent IONM did not suffer from postoperative vocal cord palsy. In contrast, patients in the mortality group without IONM developed more postoperative vocal cord palsy. We found that patients with vocal palsy had poorer 2-year Kaplan–Meier survival curves but not in patients with IONM. However, IONM was a significant predictor for less vocal cord palsy, and vocal cord palsy was also a significant predictor for poorer 2-year survival. In addition, age, advanced pathological T status, neoadjuvant chemoradiation, and prolonged ICU stay were found as predictors of a lesser 2-year survival rate. Patients who underwent neoadjuvant chemoradiation in our study had more advanced T and N status than those who underwent upfront surgery. The unequal severity and stage may contribute to the poorer survival results in this patient group.

This study has several limitations. First, there is selection bias because of its retrospective fashion and small sample size. We use IONM in patients with more advanced clinical T status, although no difference was found in most of the patient characteristics. Survival benefits may be influenced by several factors, including dose and neoadjuvant chemoradiation regimen, type of adjuvant therapy, etc. Our study revealed that IONM contributed to a better 2-year OS, but this cannot be firmly summarized in our results.

## 5. Conclusions

This study showed the feasibility and safety of intermittent IONM for bilateral RLN during esophagectomy. IONM could reduce the incidence of vocal cord palsy that resulted from RLN injury and postoperative pneumonia.

## Figures and Tables

**Table 1:** Patient characteristics.

	IONM (*n* = 38)		Non-IONM (*n* = 37)		*p*-Value
	*n* (%)		*n* (%)		
Age (mean ± SD)	60.18 ± 8.99		56.16 ± 9.78		0.068
Gender					
Male	34	89%	34	92%	0.719
Female	4	11%	3	8%	
History of H and N cancer					
No	34	89%	30	81%	0.304
Yes	4	11%	7	19%	
Neoadjuvant CCRT					
No	23	61%	17	46%	0.206
Yes	15	39%	20	54%	
cT stage					
1	6	16%	2	5%	0.038
2	9	24%	21	57%	
3	21	55%	13	35%	
4	2	5%	1	3%	
cN stage					
0	16	42%	9	24%	0.331
1	12	32%	15	41%	
2	6	16%	10	27%	
3	4	11%	3	8%	
Tumor location					
Upper thoracic/middle thoracic	27	71%	24	65%	0.566
Lower thoracic/EG junction	11	29%	13	35%	
Cell type					
Squamous cell carcinoma	33	87%	34	92%	0.716
Adenocarcinoma	4	11%	2	5%	
Others	1	3%	1	3%	

IONM, intraoperative nerve monitoring; H and N cancer, head and neck cancer; CCRT, concurrent chemoradiation therapy; EG junction, esophagogastric junction.

**Table 2:** Surgical outcome.

		IONM		Non-IONM		*p*-Value
		*n* = 38	%	*n* = 37	%	
Operation time, minutes	Median (IQR)	570	(533–644)	633	(586–688)	0.007
Blood loss, ml	Median (IQR)	100	(50–150)	150	(100–200)	0.019
ICU LOS, days	Mean ± SD	4.6 ± 2.6		5.9 ± 4.2		0.096
Hospital LOS, days	Mean ± SD	21.1 ± 9.7		19.8 ± 7.7		0.515
pT stage						
0		6	16%	10	27%	0.624
Tis		0	0%	1	3%	
1		7	18%	4	11%	
2		8	21%	5	14%	
3		16	42%	16	43%	
4		1	3%	1	3%	
pN stage						
0		19	50%	17	46%	0.337
1		8	21%	13	35%	
2		11	29%	7	19%	
Harvested MLN		30.0 ± 12.9		28.9 ± 8.7		0.670
Positive MLN		1.0 ± 1.6		0.7 ± 1.0		0.391
Ratio of positive MLN		2.97%		2.53%		
Harvested TLN		41.9 ± 13.9		41.5 ± 11.7		0.898
Positive TLN		1.4 ± 1.8		1.3 ± 1.7		0.710
Ratio of positive TLN		3.23%		3.01%		
Harvested right RLN LN		3.6 ± 2.4		3.3 ± 2.3		0.572
Harvested left RLN LN		3.1 ± 3.2		1.8 ± 2.0		0.043

IONM, intraoperative nerve monitoring; IQR, interquartile range; LOS, length of stay; MLN, mediastinal lymph nodes; TLN, total lymph nodes; RLN, recurrent laryngeal nerve; LN, lymph nodes.

**Table 3:** Postoeprative morbidity.

	IONM		non-IONM		*p*-Value
	*n*	%	*n*	%	
Vocal cord palsy	4	10.5%	14	37.8%	0.006
Right/left/bilateral	2/2/0		3/10/1		
Pneumonia	5	13.2%	14	37.8%	0.014
Other complications					
Anastomosis leakage	5	28%	3	14%	0.298
Pneumothorax/air leak	1	6%	1	5%	0.911
Chylothorax	2	11%	0	0%	0.117
Chyloabdomen	1	6%	1	5%	0.911
Loculated pleural effusion	3	17%	2	10%	0.506
Respiratory failure	0	0%	1	5%	0.348

IONM, intraoperative nerve monitoring.

**Table 4:** Hazard ratios for death (analysis from time of surgery) using the backward elimination (BE).

	HR	95.0% CI	*p*-Value
Vocal cord palsy (with)	2.98	(1.32–6.71)	0.008
Age (year)	1.05	(1.01–1.08)	0.016
pT(Tis + T1a/b + T2)	7.25	(2.09–25.17)	0.002
pT(T3 + T4)	12.53	(3.62–43.35)	0.000
Neoadjuvant CCRT	8.37	(3.59–19.52)	0.000
ICU duration (day)	1.11	(1.00–1.22)	0.040

HR, hazard ratio; CI, confidence interval

## Data Availability

Not applicable.

## References

[1] Sung H., Ferlay J., Siegel R.L., Laversanne M., Soerjomataram I., Jemal A., Bray F. (2021). Global Cancer Statistics 2020: GLOBOCAN Estimates of Incidence and Mortality Worldwide for 36 Cancers in 185 Countries. CA Cancer J. Clin..

[2] Demarest C.T., Chang A.C. (2021). The Landmark Series: Multimodal Therapy for Esophageal Cancer. Ann. Surg. Oncol..

[3] Akiyama H., Tsurumaru M., Udagawa H., Kajiyama Y. (1994). Radical lymph node dissection for cancer of the thoracic esophagus. Ann Surg..

[4] Bona D., Lombardo F., Matsushima K., Cavalli M., Lastraioli C., Bonitta G., Cirri S., Danelli P., Aiolfi A. (2022). Three-field versus two-field lymphadenectomy for esophageal squamous cell carcinoma: A long-term survival meta-analysis. Surgery.

[5] Kang C.H., Kim Y.T., Jeon S.H., Sung S.W., Kim J.H. (2007). Lymphadenectomy extent is closely related to long-term survival in esophageal cancer. Eur. J. Cardiothorac. Surg..

[6] Deng H.Y., Wang Z.Q., Wang Y.C., Li G., Luo J., Chen L.Q., Liu L.X., Zhou Q.H., Lin Y.D. (2017). Oesophageal adenocarcinoma has a higher risk of lymph node metastasis than squamous cell carcinoma: A propensity score-matched study. Eur. J. Cardiothorac. Surg..

[7] Mine S., Sano T., Hiki N., Yamada K., Kosuga T., Nunobe S., Shigaki H., Yamaguchi T. (2014). Thoracic lymph node involvement in adenocarcinoma of the esophagogastric junction and lower esophageal squamous cell carcinoma relative to the location of the proximal end of the tumor. Ann. Surg. Oncol..

[8] Mine S., Watanabe M., Kumagai K., Okamura A., Yuda M., Hayami M., Yamashita K., Imamura Y., Ishizuka N. (2019). Comparison of mediastinal lymph node metastases from adenocarcinoma of the esophagogastric junction versus lower esophageal squamous cell carcinoma with involvement of the esophagogastric junction. Dis. Esophagus.

[9] Kanemura T., Makino T., Miyazaki Y., Takahashi T., Kurokawa Y., Yamasaki M., Nakajima K., Takiguchi S., Mori M., Doki Y. (2017). Distribution patterns of metastases in recurrent laryngeal nerve lymph nodes in patients with squamous cell esophageal cancer. Dis. Esophagus.

[10] Ma L., Xiang J., Zhang Y., Hu H., Shao R., Lin D. (2017). Characteristics and clinical significance of recurrent laryngeal nerve lymph node metastasis in esophageal squamous cell carcinoma. J. BUON.

[11] Jang H.J., Lee H.S., Kim M.S., Lee J.M., Zo J.I. (2011). Patterns of lymph node metastasis and survival for upper esophageal squamous cell carcinoma. Ann. Thorac. Surg..

[12] Baba M., Natsugoe S., Shimada M., Nakano S., Noguchi Y., Kawachi K., Kusano C., Aikou T. (1999). Does hoarseness of voice from recurrent nerve paralysis after esophagectomy for carcinoma influence patient quality of life?. J. Am. Coll. Surg..

[13] Shimizu H., Shiozaki A., Fujiwara H., Konishi H., Kosuga T., Komatsu S., Ichikawa D., Okamoto K., Otsuji E. (2017). Short- and Long-term Progress of Recurrent Laryngeal Nerve Paralysis After Subtotal Esophagectomy. Anticancer Res..

[14] Oshikiri T., Takiguchi G., Hasegawa H., Yamamoto M., Kanaji S., Yamashita K., Matsuda T., Nakamura T., Suzuki S., Kakeji Y. (2021). Postoperative recurrent laryngeal nerve palsy is associated with pneumonia in minimally invasive esophagectomy for esophageal cancer. Surg. Endosc..

[15] Wright C.D., Zeitels S.M. (2006). Recurrent laryngeal nerve injuries after esophagectomy. Thorac. Surg. Clin..

[16] Yang S., Zhou L., Lu Z., Ma B., Ji Q., Wang Y. (2017). Systematic review with meta-analysis of intraoperative neuromonitoring during thyroidectomy. Int. J. Surg..

[17] Ratner B. (2010). Variable selection methods in regression: Ignorable problem, outing notable solution. J. Target Meas. Anal. Mark..

[18] Zhong D., Zhou Y., Li Y., Wang Y., Zhou W., Cheng Q., Chen L., Zhao J., Li X., Yan X. (2014). Intraoperative recurrent laryngeal nerve monitoring: A useful method for patients with esophageal cancer. Dis. Esophagus.

[19] Hikage M., Kamei T., Nakano T., Abe S., Katsura K., Taniyama Y., Sakurai T., Teshima J., Ito S., Niizuma N. (2017). Impact of routine recurrent laryngeal nerve monitoring in prone esophagectomy with mediastinal lymph node dissection. Surg. Endosc..

[20] Takeda S., Iida M., Kanekiyo S., Nishiyama M., Tokumitsu Y., Shindo Y., Yoshida S., Suzuki N., Yoshino S., Nagano H. (2021). Efficacy of intraoperative recurrent laryngeal neuromonitoring during surgery for esophageal cancer. Ann. Gastroenterol. Surg..

[21] Fan N., Yang H., Zheng J., Chen D., Wang W., Tan Z., Huang Y., Lin P. (2019). Comparison of short- and long-term outcomes between 3-field and modern 2-field lymph node dissections for thoracic oesophageal squamous cell carcinoma: A propensity score matching analysis. Interact. Cardiovasc. Thorac. Surg..

[22] Hemmerling T.M., Schmidt J., Jacobi K.E., Klein P. (2001). Intraoperative monitoring of the recurrent laryngeal nerve during single-lung ventilation in esophagectomy. Anesth. Analg..

[23] Schmidt J., Irouschek A., Heinrich S., Oster O., Klein P., Birkholz T. (2012). Recurrent laryngeal nerve monitoring during esophagectomy and mediastinal lymph node dissection: A novel approach using a single-lumen endotracheal EMG tube and the EZ-blocker. World J. Surg..

[24] Gelpke H., Grieder F., Decurtins M., Cadosch D. (2010). Recurrent laryngeal nerve monitoring during esophagectomy and mediastinal lymph node dissection. World J. Surg..

